# Hydrogen-Rich Water Intake Accelerates Oral Palatal Wound Healing via Activation of the Nrf2/Antioxidant Defense Pathways in a Rat Model

**DOI:** 10.1155/2016/5679040

**Published:** 2015-12-21

**Authors:** Naofumi Tamaki, Rita Cristina Orihuela-Campos, Makoto Fukui, Hiro-O Ito

**Affiliations:** Department of Preventive Dentistry, Institute of Biomedical Sciences, Tokushima University Graduate School, 3-18-15 Kuramoto-cho, Tokushima 770-8504, Japan

## Abstract

The wound healing process attempts to restore the integrity and function of the injured tissue. Additionally, proinflammatory cytokines, growth factors, and oxidative stress play important roles in wound healing. The aim of this study was to determine whether hydrogen-rich water intake induces the activation of the Nrf2/antioxidant defense pathway in rat palatal tissue, thereby reducing systemic oxidative stress and proinflammatory cytokine levels and promoting healing-associated genes. A circular excisional wound was created in the oral palatal region, and the wound healing process was observed. The rats were divided into two experimental groups in which either hydrogen-rich water or distilled water was consumed. In the drinking hydrogen-rich water, the palatal wound healing process was accelerated compared to that in the control group. As molecular hydrogen upregulated the Nrf2 pathway, systemic oxidative stresses were decreased by the activation of antioxidant activity. Furthermore, hydrogen-rich water intake reduced proinflammatory cytokine levels and promoted the expression of healing-associated factors in rat palatal tissue. In conclusion, hydrogen-rich water intake exhibited multiple beneficial effects through activation of the Nrf2/antioxidant defense pathway. The results of this study support the hypothesis that oral administration of hydrogen-rich water benefits the wound healing process by decreasing oxidative stress and inflammatory responses.

## 1. Introduction

The wound healing process following trauma-related injury is involved in restoration of the integrity and function of the injured tissue. This process initiates an orderly but complex sequence of events that establish the integrity of the damaged tissues. Regeneration is considered to have taken place when tissue is structurally and functionally repaired to its original state as a result of the healing process. In general, wound healing occurs in a cascade of overlapping phases, beginning with the inflammatory phase precipitated by the injury and followed sequentially by the proliferation and tissue remodeling phases [1]. Multiple proinflammatory cytokines, chemokines, and growth factors contribute to the success of wound repair. In addition, it is also important to focus on reactive oxygen species (ROS) in the context of wound healing [2].

Oxidative stress has been reported in many diseases in which the production of ROS and the oxidative modification of various biomolecules underlie their pathophysiology, especially inflammation-associated processes. There is evidence that oxidative stress is a key factor contributing to inflammatory conditions such as recurrent aphthous stomatitis, which causes a wound in the oral mucosa with no well-established underlying cause [3]. During wound-induced inflammation, ROS and inflammatory cytokines are released from immune system cells to kill microorganisms. However, these molecules are also known to be a significant factor in the etiology of local tissue damage [4]. The production of ROS in the inflammatory process leads to marked inflammation during which the secretion of various chemoattractants is noted. Chemotactic stimulation of phagocytosis induces the activation of a membrane-bound enzyme system that transfers electrons from cytosolic nicotinamide adenine dinucleotide phosphate (NADP^+^) to extracellular oxygen, resulting in the production of the superoxide radical, a well-established ROS [5].

The beneficial biological effects of molecular hydrogen in many diseases and physiological states are overwhelmingly supported in relevant scientific literature [6]. Molecular hydrogen has potential as a novel antioxidant in preventive and therapeutic applications, in addition to its anti-inflammatory effects [7]. It has also been reported that molecular hydrogen activates the nuclear factor-E2 related factor 2 (Nrf2)/antioxidant defense pathway [8]. The translocation of Nrf2 into the nucleus leads to upregulation of the expression of genes encoding phase 2 enzymes involved in defense systems against oxidative stress and other toxic sources [9]. Nrf2 cooperates with the antioxidant defense system to regulate genes such as heme oxygenase 1 (*HO-1*) and NAD(P)H quinine oxidoreductase 1 (*NQO-1*).

It has been reported that the inhalation of hydrogen-containing gas stimulates the healing of radiation-induced skin wounds [10]. However, it is unclear whether the benefits of hydrogen-rich water intake on oxidative stress and inflammation are involved in the healing of oral palatal wounds. The objective of this study was to determine whether hydrogen-rich water induces the activation of the Nrf2/antioxidant defense pathway in palatal tissue, thereby reducing oxidative stress and proinflammatory cytokine levels. In a rat model, we examined whether these effects could promote the wound healing process. Wound closure was monitored and gene expression determined in rat palatal tissue after injury for proinflammatory cytokines (interleukin 1 beta [IL-1*β*], interleukin 6 [IL-6], and tumor necrosis factor alpha [TNF-*α*]); chemokines (monocyte chemotactic protein-1 [MCP-1] and macrophage inflammatory protein-1 alpha [MIP-1*α*]); growth factors (transforming growth factor beta 1 [TGF-*β*1], fibroblast growth factor 7 [FGF7], and vascular endothelial growth factor [VEGF]); and healing-associated factors (alpha smooth muscle actin [*α*-SMA] and type 1 collagen [Col-1]). All of these genes are important for the wound healing process and show high levels of expression early in the remodeling phase.

## 2. Material and Methods

### 2.1. Animals

Twenty-four male Wistar rats (8 weeks of age) weighing 320–340 g were housed in individual wire cages in a temperature- and humidity-controlled room (23°C ± 1°C and 60% ± 5% relative humidity) with a 12 h light-dark cycle. The animals were given standard rat pellets and drinking water. All the animals received humane care in compliance with institutional animal care guidelines, and the Animal Research Control Committee of Tokushima University Graduate School (protocol number 12098) approved the experimental protocol.

### 2.2. Wounding and Preparation of Wound Tissue

**Figure 1:**
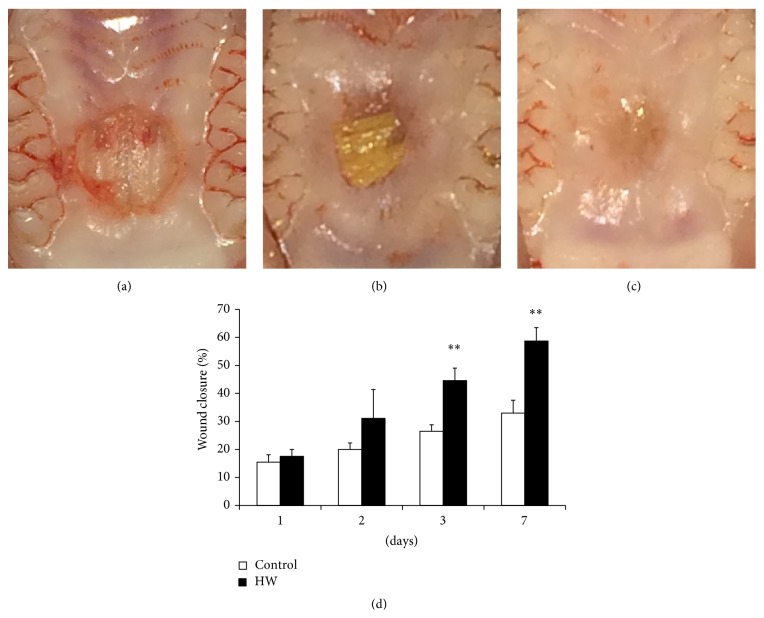
Photographs and percentage of healing of the palatal wounds in rats drinking either distilled or hydrogen-rich water. (a) A circular (3.5 mm in diameter) full-thickness excision wound was created with a surgical punch at baseline. The photographs of palatal wounds of (b) control group and (c) HW group at 7 days after treatment are shown. (d) Changes in palatal wound closure for each group were calculated and plotted over the course of this study. Comparisons are represented within each time point. Data are expressed as mean ± SEM (*n* = 6). ^*∗∗*^ *p* < 0.01: significantly different from control group.

Prior to wounding, all rats were anesthetized with an injection of sodium pentobarbital with physiological saline, after which a circular full-thickness excisional wound, 3.5 mm in diameter, was created using a sterile biopsy punch (Kai Medical, Seki City, Japan) in the center of the oral palatal region in each animal (Figure 1(a)) [11]. The animals were observed and weighed on days 0, 1, 2, 3, and 7. For the purpose of observation, rats were anesthetized using an inhalational anesthetic with isoflurane; clinical photographs of palatal wounds were taken with a digital camera on days 0, 1, 2, 3, and 7. In each photograph, a stainless steel ruler was included to provide calibration of the wound area measurement between images [2]. A free computer program (Image J; NIH, Bethesda, MD, USA) was used to measure the wound area. The palatal wound closure of each time point was calculated using the following formula: percentage of wound closure (%) = (WA_0_ − WA_*t*_)/WA_0_ × 100, where WA corresponds to wound area, 0 means day 0 (baseline), and *t* represents each time point after treatment.

The rats were randomly divided into two experimental groups of twelve each: (1) control group treated with distilled water and (2) hydrogen water group (HW) treated with hydrogen-rich water. Each type of water was placed twice a day in a closed glass vessel equipped with an outlet line containing two ball bearings, which prevented the water from being degassed. The molecular hydrogen-preserving capacity of this method has previously been established [12]. The rats consumed distilled water or hydrogen-rich water until 3 or 7 days after treatment.

Six rats from each group were sacrificed under general anesthesia, and cardiac blood samples were collected from the heart at each experimental time point: 3 or 7 days after treatment. Serum was separated by centrifugation at 3,000 ×g for 20 min and stored at −80°C until use. The entire wound area was harvested using a sterile biopsy punch from six rats per group on day 3 or day 7. The excised palatal tissue was immediately subdivided and processed for use in each experiment [13].

### 2.3. Preparation of Hydrogen-Rich Water

Hydrogen-rich water was prepared using an Aquela kit (Ecomo International Co., Ltd., Fukuoka, Japan). Hydrogen gas was obtained in an acrylic resin tube by using material for producing molecular hydrogen in a polyethylene terephthalate (PET) bottle so as to provide a carbonated drink containing 500 mL water [14]. The concentration of molecular hydrogen in the water was measured according to a previously reported method [15]. We confirmed that the molecular hydrogen concentration was 5–7 ppm in hydrogen-rich water before use. In a glass vessel, the hydrogen concentration was more than 1 ppm after 24 h.

### 2.4. Real-Time PCR

The divided palatal samples were immediately placed in 1 mL RNAlater (Sigma, St. Louis, MO, USA) and frozen. Total RNA from a palatal tissue specimen was isolated using TRIzol (Invitrogen Corporation, Grand Island, NY, USA) according to the manufacturer's protocol. The RNA concentration was determined from the optical density at a wavelength of 260 nm using an OD_260_ unit equivalent to 40 *μ*g/mL of RNA. The purity of the isolated RNA was determined by the absorbance ratio of 260/280 nm, and only samples with a ratio of more than 1.8 were used. One microgram of isolated total RNA was reverse transcribed to cDNA using ReverTra Ace (Toyobo Co., Ltd., Osaka, Japan).  Table 1 displays the primer sequences for the genes of interest in this study. Quantification was performed using a real-time PCR system (Bio-Rad, Hercules, CA, USA) with SYBR Green (Bio-Rad) [16]. The cycling parameters were as follows: initial denaturation at 95°C for 1 min, followed by 40 cycles of denaturation at 95°C for 20 s, annealing at 60°C for 30 s, and extension at 72°C for 30 s. The mRNA levels were calculated by determining the relative copy number of each mRNA to that of the housekeeping internal control gene (GAPDH) for each sample, and *C*_*t*_ of the control group at 3 days after treatment was normalized to 1.

### 2.5. Western Blot Analysis

The divided rat palatal tissue was harvested in lysis buffer, to which a protease inhibitor cocktail was added and thoroughly homogenized. Protein content was determined with Bradford reagent using bovine serum albumin as a standard. An equal amount of protein from palatal tissue was separated by sodium dodecyl sulphate polyacrylamide gel electrophoresis for 30 min at 200 V and was transferred onto polyvinylidene difluoride membranes for 60 min at 100 V. Following transfer, the membranes were blocked with 5% nonfat dry milk in Tris-buffered saline with Tween-20 for 1 h. Subsequently, the membranes were probed with primary antibody (iNOS [1 : 200 dilution; Santa Cruz Biotechnology, Santa Cruz, CA, USA]) and *β*-actin (1 : 1000 dilution; Cell Signaling Technology, Danvers, MA, USA) overnight at 4°C, followed by incubation with an anti-mouse horseradish peroxidase-conjugated secondary antibody (1 : 3000 dilution; Cell Signaling Technology) for 1 h. Detection was performed using enhanced chemiluminescence. Immunoblots were scanned by densitometry and the intensity was quantified using Image J software (NIH).

### 2.6. Serum Levels of Reactive Nitrogen Species Biomarkers

Nitric oxide (NO) metabolite (NOx; nitrite + nitrate) levels were determined with a nitrite/nitrate colorimetric assay kit using the Griess reaction (Dojindo, Tokyo, Japan), according to the manufacturer's instructions. NOx concentrations have been expressed in *μ*mol/L [17]. The levels of serum nitrotyrosine were determined using an ELISA kit (Immundiagnostik AG, Bensheim, Germany) [18]. Nitrotyrosine concentrations have been expressed in nM.

### 2.7. Serum Levels of Oxidative Stress

Measurement of serum reactive oxygen metabolites (ROMs) levels was performed using photometric quantification as previously reported [19], according to the manufacturer's instructions (Diacron International, Grosseto, Italy). ROM tests are expressed in relative units, where one Carratelli unit (CARR U) is equivalent to 0.08 mg/dL of a hydrogen peroxide solution. To determine total serum antioxidant capacity, the OXY-adsorbent tests were performed using a spectrophotometer (Diacron International) for all samples [19]. This test evaluates the capacity of serum to oppose the massive oxidative action of a hypochlorous acid (HClO) solution; total antioxidant capacity was expressed in terms of HClO consumed by 1 mL of sample (l mol HClO/mL).

### 2.8. Serum Levels of Proinflammatory Cytokines

All serum samples analyzed for proinflammatory cytokines were filtered through a 0.22 *μ*m spin filter. Multiplex bead-based kits (Bio-Rad) were used to measure the concentrations of proinflammatory cytokines (IL-1*β*, IL-6, and TNF-*α*) in serum. The cytokines were measured in a 96-well plate using 50 *μ*L of serum per well. The standards and samples were assayed on a Bio-Plex 200 system (Bio-Rad); plates were washed with the Bio-Plex Pro wash station (Bio-Rad) for magnetic beads as reported earlier [20]. Samples were analyzed and standard curves were generated using the Bio-Plex Manager version 5.0 software (Bio-Rad).

### 2.9. Statistical Analysis

All data were expressed as mean ± standard error of the mean (SEM). For statistical analysis, differences between groups were analyzed with the Mann-Whitney *U* test using IBM SPSS statistics version 19 (SPSS Inc., Tokyo, Japan). A *p* value less than 0.05 was considered statistically significant. A sample size of 6 per group was required to detect significant differences based on the results of a previous study [21].

## 3. Results

### 3.1. Palatal Wound Closure and General Characteristics

There were no significant differences between the groups with respect to food consumption, body weight, or growth patterns during the experimental period. Photographs of palatal wound creation and healing in rats are shown in Figures 1(a), 1(b), and 1(c). In this model, clinical examination of wounds showed gradual healing in a time-dependent manner in all groups. The time course of palatal wound closure for each group at 1, 2, 3, and 7 days after treatment is shown in Figure 1(d). There were no significant differences between the groups at 1 and 2 days. However, the rates of wound closure of the HW group were significantly higher than those of the control group at both 3 and 7 days after treatment (*p* < 0.01). Acceleration of the wound healing process was observed in the HW group, which consumed hydrogen-rich water.

### 3.2. Effects of Hydrogen-Rich Water on the Nrf2/Antioxidant Defense Pathway

**Figure 2:**
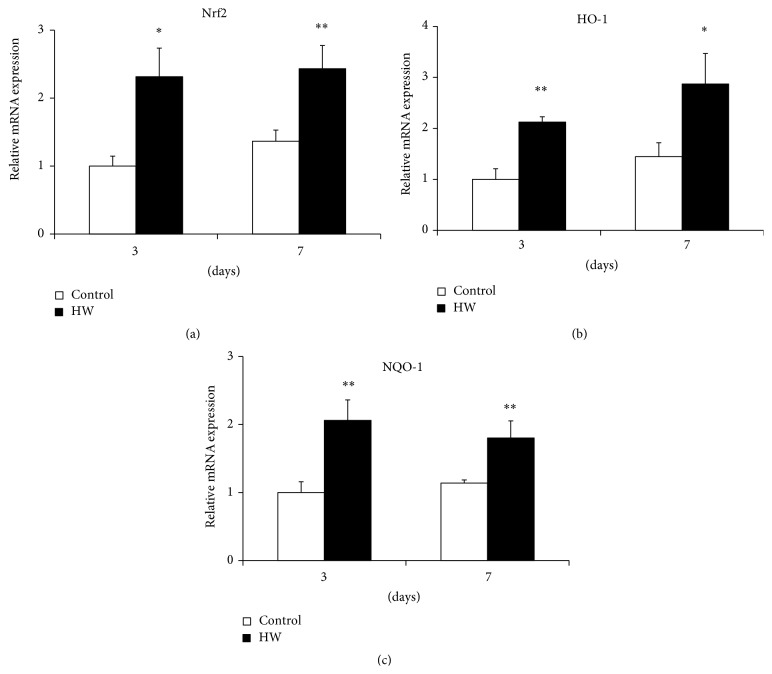
Effects of hydrogen-rich water on the expression of genes involved in the Nrf2/antioxidant defense pathway in rat palatal tissue. Relative mRNA levels of (a) Nrf2, (b) HO-1, and (c) NQO-1 were determined by real-time PCR. Bars represent mRNA expression levels normalized to GAPDH levels and relative to the control group at 3 days. Data are expressed as mean ± SEM (*n* = 6). ^*∗*^ *p* < 0.05, ^*∗∗*^ *p* < 0.01: significantly different from control group at each time point.

The Nrf2 mRNA expression levels were significantly higher in rat palatal tissue with hydrogen-rich water intake than in palatal tissue of the control group (*p* < 0.05; *p* < 0.01) (Figure 2(a)). The HW group at 7 days after treatment displayed a further increase in Nrf2 expression versus 3 days (Figure 2(a)). Additionally, the mRNA expression of HO-1 had significantly increased at both 3 and 7 days after treatment (*p* < 0.05; *p* < 0.01) (Figure 2(b)). The mRNA expression levels of NQO-1, which is known to be induced by Nrf2, were markedly higher in the HW group than in the control group at both 3 and 7 days (*p* < 0.01) (Figure 2(c)). These data suggest that hydrogen-rich water intake promoted the upregulation of the expression of antioxidant defense genes by activating the Nrf2 pathway.

### 3.3. Effects of Hydrogen-Rich Water on iNOS in Rat Palatal Tissue

**Figure 3:**
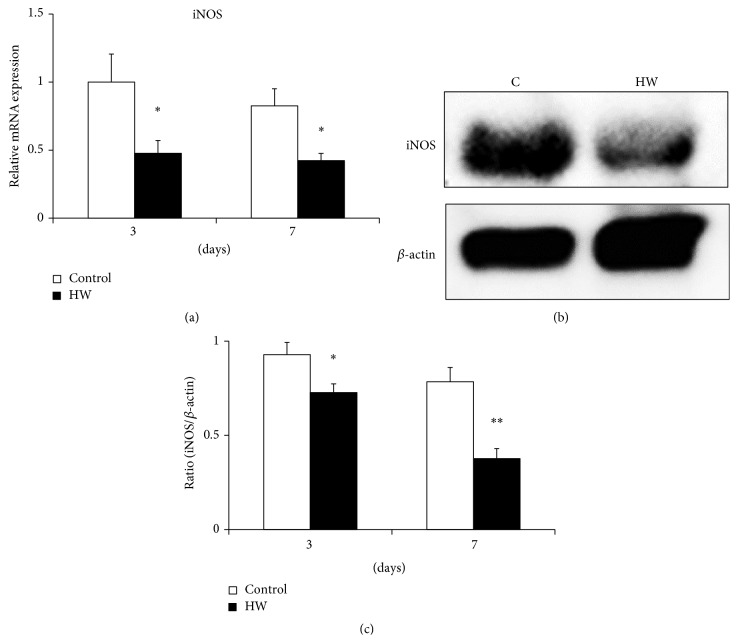
Effects of hydrogen-rich water on iNOS expression in rat palatal tissue. (a) Relative mRNA levels of iNOS were detected by real-time PCR. Bars represent mRNA expression levels normalized to GAPDH levels and relative to the control group at 3 days. (b) Representative western blots are shown for iNOS and *β*-actin. (c) Densitometry results for the bands (iNOS) were normalized to those for *β*-actin. Data are expressed as mean ± SEM (*n* = 6). ^*∗*^ *p* < 0.05, ^*∗∗*^ *p* < 0.01: significantly different from control group at each time point.

The mRNA expression of iNOS in rats with hydrogen-rich water intake was lower than that in the control group at both 3 and 7 days after treatment (*p* < 0.05) (Figure 3(a)). Western blot analysis indicated that iNOS protein expression was attenuated in the HW group (Figure 3(b)). Furthermore, rats in the HW group exhibited significantly suppressed iNOS protein expression at both 3 and 7 days after treatment (*p* < 0.05; *p* < 0.01) (Figure 3(c)). Taken together, the results indicate that activation of the Nrf2/antioxidant defense pathway reduced iNOS expression in rat palatal tissue.

### 3.4. Levels of Nitrosative and Oxidative Stress Biomarkers in Serum

**Figure 4:**
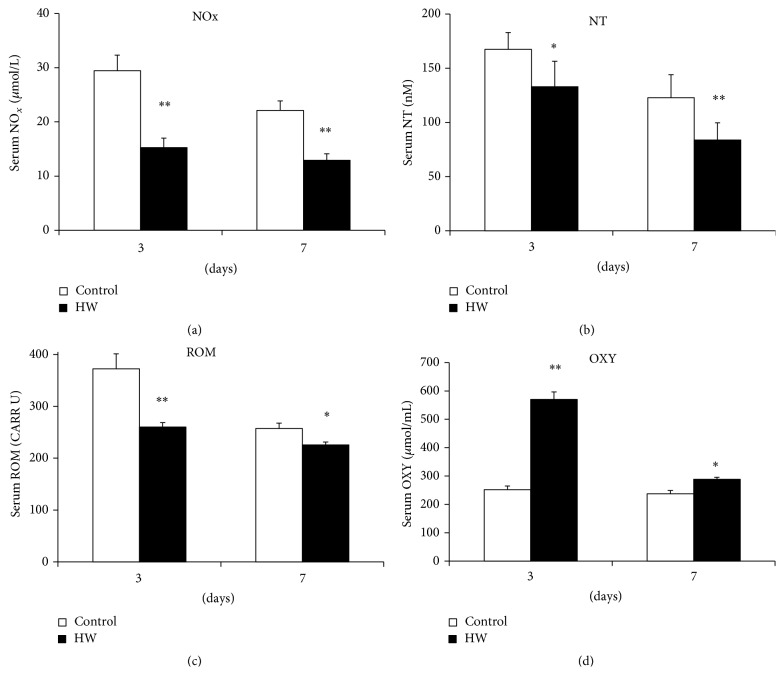
Levels of nitrosative and oxidative stress biomarkers in serum. (a) NOx was quantified by the nitrite/nitrate colorimetric assay using the Griess reaction and (b) nitrotyrosine (NT) was quantified by ELISA. (c) ROM and (d) OXY were quantified using photometric quantification. Data are expressed as means ± SEM (*n* = 6). ^*∗*^ *p* < 0.05, ^*∗∗*^ *p* < 0.01: significantly different from control group at each time point.

We assessed whether hydrogen-rich water intake regulated systemic nitrosative stress. Levels of nitrosative stress biomarkers such as NOx were markedly lower in rats with hydrogen-rich water intake than in control rats at both 3 and 7 days after treatment (*p* < 0.01) (Figure 4(a)). Moreover, serum levels of nitrotyrosine significantly decreased in the HW group at 3 and 7 days (*p* < 0.05; *p* < 0.01) (Figure 4(b)).

To evaluate the effects of hydrogen-rich water on systemic oxidative stress, we measured biomarkers of oxidative stress, such as ROM and OXY. Serum levels of ROM, a reliable biomarker of circulating ROS, were significantly lowered by intake of hydrogen-rich water at 3 and 7 days after treatment (*p* < 0.05; *p* < 0.01) (Figure 4(c)). Moreover, levels of antioxidant capacity, such as OXY in the HW group, were markedly higher than those of the control group at 3 and 7 days after treatment (*p* < 0.05; *p* < 0.01) (Figure 4(d)). These results indicate that hydrogen-rich water intake reduced systemic nitrosative and oxidative stresses by the activation of antioxidant activity.

### 3.5. Effects of Hydrogen-Rich Water on Proinflammatory Cytokines and Chemokines in Rat Palatal Tissue

**Figure 5:**
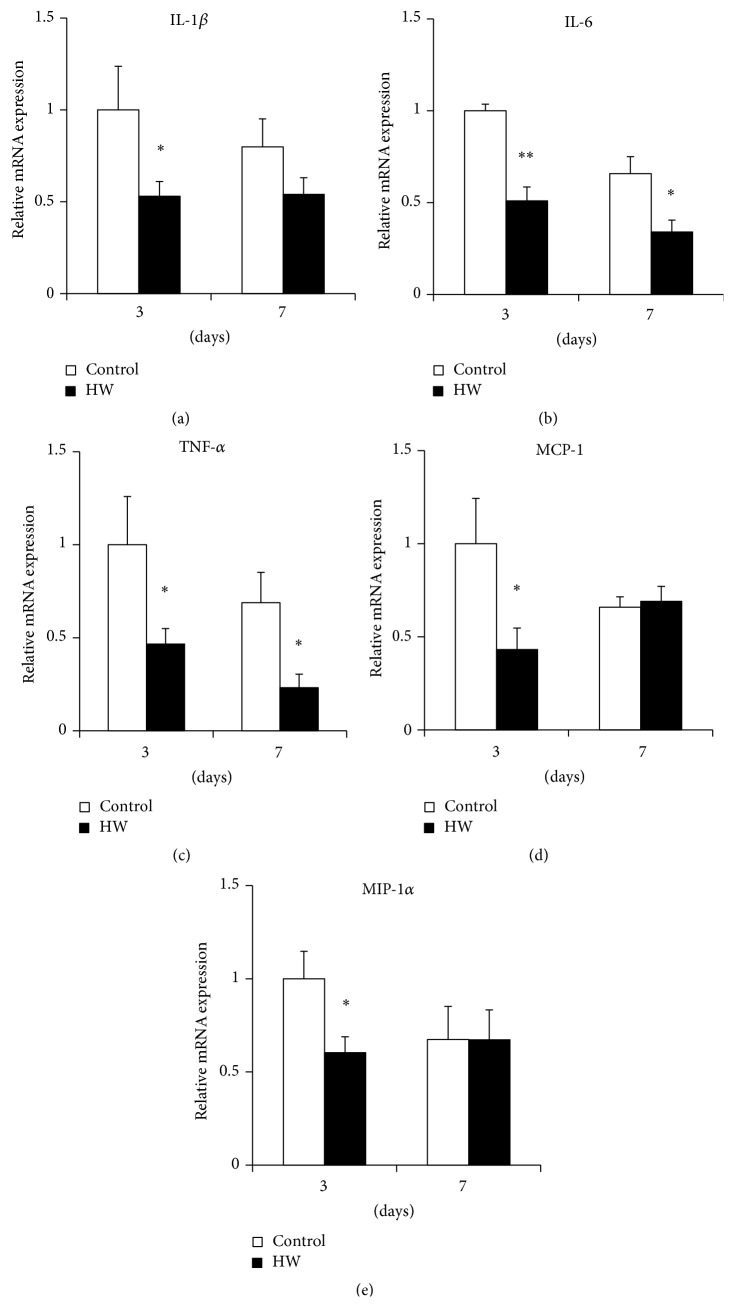
Effects of hydrogen-rich water on proinflammatory cytokines and chemokine expression in rat palatal tissue. Relative mRNA levels were detected for (a) IL-1*β*, (b) IL-6, (c) TNF-*α*, (d) MCP-1, and (e) MIP-1*α* by real-time PCR. Bars represent mRNA expression levels normalized to GAPDH levels and relative to the control group at 3 days after treatment. Comparisons within each time point at 3 and 7 days are shown. Data are expressed as mean ± SEM (*n* = 6). ^*∗*^ *p* < 0.05, ^*∗∗*^ *p* < 0.01: significantly different from control group at each time point.

Using real-time PCR, we found that the HW group had lower proinflammatory cytokine expression levels in rat palatal tissue than the control group (Figures 5(a), 5(b), and 5(c)). Specifically, the expression of IL-6 and TNF-*α* was significantly lower at 3 and 7 days after treatment (*p* < 0.05; *p* < 0.01) (Figures 5(b) and 5(c)). In contrast, the expression levels of chemokines in the HW group were only significantly suppressed at 3 days (*p* < 0.05) (Figures 5(d) and 5(e)). These results indicate that hydrogen-rich water intake suppressed the expression of inflammatory cytokines and chemokines.

### 3.6. Effects of Hydrogen-Rich Water on the Expression of Healing-Associated Genes in Rat Palatal Tissue

**Figure 6:**
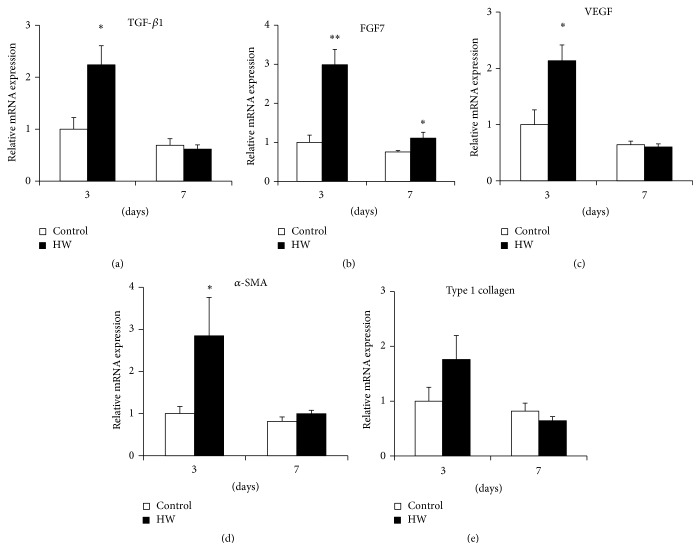
Effects of hydrogen-rich water on the expression of healing-associated genes in rat palatal tissue. Relative mRNA levels were detected for (a) TGF-*β*1, (b) FGF7, (c) VEGF, (d) *α*-SMA, and (e) type 1 collagen by real-time PCR. Bars represent mRNA expression levels normalized to GAPDH levels and relative to the control group at 3 days after treatment. Comparisons within each time point at 3 and 7 days are shown. Data are expressed as mean ± SEM (*n* = 6). ^*∗*^ *p* < 0.05, ^*∗∗*^ *p* < 0.01: significantly different from control group at each time point.

The gene expression of TGF-*β*1, FGF7, VEGF, and *α*-SMA was higher in the HW group than the control group at 3 days after treatment (*p* < 0.05) (Figures 6(a), 6(b), 6(c), and 6(d)). The HW group exhibited significantly upregulated expression of FGF7 mRNA as compared to that in the periodontitis group at both 3 and 7 days after treatment (*p* < 0.05; *p* < 0.01) (Figure 6(b)). Although no significant difference in type 1 collagen levels was observed between the groups, borderline significantly higher levels were observed in the HW group at 3 days (Figure 6(e)). These data suggest that hydrogen-rich water intake promotes the expression of healing-associated genes in rat palatal tissue.

### 3.7. Effects of Hydrogen-Rich Water on Systemic Levels of Proinflammatory Cytokines

**Figure 7:**
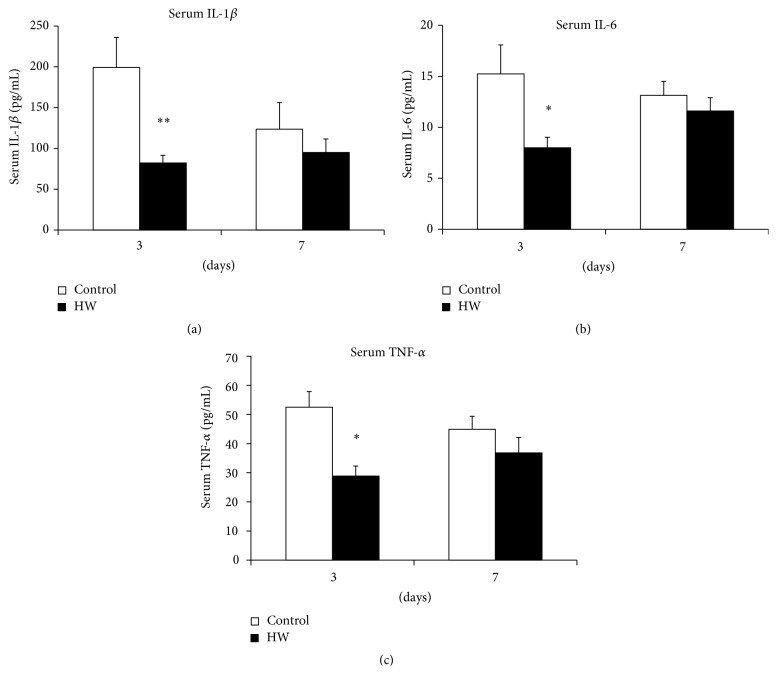
Effects of hydrogen-rich water on proinflammatory cytokine concentrations in serum. (a) IL-1*β*, (b) IL-6, and (c) TNF-*α* were quantified with a multiplex bead assay. Data are expressed as mean ± SEM (*n* = 6). ^*∗*^ *p* < 0.05, ^*∗∗*^ *p* < 0.01: significantly different from control group at each time point.

The serum levels of the proinflammatory cytokines IL-1*β*, IL-6, and TNF-*α* were analyzed using a multiplex bead-based system. In rats drinking hydrogen-rich water, serum IL-1*β*, IL-6, and TNF-*α* levels were significantly lower than those of the control group at 3 days after treatment (*p* < 0.05; *p* < 0.01) (Figure 7). However, no significant differences in IL-1*β*, IL-6, and TNF-*α* levels were observed between the groups; a trend toward lower levels was observed in the HW group at 7 days (Figure 7). These data suggest that hydrogen-rich water lowers systemic levels of proinflammatory cytokines.

## 4. Discussion

The palatal excisional used to wound rats in this study was chosen for its clinical reproducibility. Many studies used this model to investigate intraoral wound healing or factors that could affect it. Furthermore, we showed that the wound healing process was accelerated by the administration of hydrogen-rich water. To elucidate the mechanisms of wound healing, we focused on the antioxidant and anti-inflammatory effects of molecular hydrogen in the rat palatal wound model. Hydrogen-rich water intake promoted the upregulation of antioxidant defense genes through activation of the Nrf2 pathway and the consequent reduced iNOS expression in palatal tissue. Systemic oxidative and nitrosative stresses decreased because of activation of the antioxidant activity by molecular hydrogen. Our findings also indicated that hydrogen-rich water intake reduced local and systemic proinflammatory cytokine levels and promoted the expression of healing-associated genes in the rat palatal tissue. Taken together, these results demonstrate that molecular hydrogen intake can promote the oral wound healing process in rats by activating the Nrf2/antioxidant defense pathway.

Wound healing is known to be a highly ordered and well-coordinated process that involves inflammation, cell proliferation, matrix deposition, and tissue remodeling [22]. The inflammatory phase is characterized by the formation of a blood clot caused by the disruption of blood vessels and extravasation of blood constituents. In addition, chemokine family members, including MCP-1 and MIP-1*α*, play the important role of mediating infiltration of inflammatory cells such as monocytes and macrophages into healing wounds [23]. TGF-*β* signaling also plays an important role during the wound healing process. TGF-*β*1 was found to facilitate the expression of *α*-SMA, a major mediator of wound contraction [24]. Since TGF-*β*1 signaling has been implicated in collagen deposition, TGF-*β*1 is also important in multiple aspects of wound healing, including epithelial growth, vascular endothelial growth, and collagen deposition [25]. FGF7 is known to regulate genes that encode cytoprotective mediators [26] and the transcription factor Nrf2, in addition to the antioxidant enzyme gene targets of Nrf2 [27]. Interestingly, a recombinant form of FGF7 is used clinically in the treatment of oral mucositis [28]. These results were consistent with previously mentioned findings; hydrogen-rich water intake suppressed the expression of the proinflammatory cytokines MCP-1 and MIP-1, whereas it enhanced the expression of healing-associated genes, such as TGF-*β*1, FGF7, VEGF, and *α*-SMA in this study.

Molecular hydrogen is the simplest element in nature and is of therapeutic and preventive interest because of its biological activities, including its antioxidant and anti-inflammatory activities in most tissues of model animals [9]. There are a number of advantages of hydrogen as a potential antioxidant: it is mild enough not to disturb metabolic redox reactions or to affect ROS, which functions in cell signaling, and it also has favorable distribution characteristics with respect to its physical ability to penetrate biomembranes and diffuse through barriers into cellular components [6]. Molecular hydrogen has also been reported to reduce the levels of proinflammatory cytokines and suppress inflammation in many experimental model animals [9]. Recently, hydrogen-rich water, a colorless and odorless liquid, was found to possess strong antioxidant and anti-inflammatory activity [29]. The main concept behind using hydrogen-rich water to promote wound healing in this study is that molecular hydrogen reduces ROS levels and inflammation. Our present study indicates that oral administration of hydrogen-rich water has beneficial effects in a rat palatal wound model. It is conceivable that orally administered molecular hydrogen could improve local redox balance as well as decrease circulating oxidative stress.

Although there are several ways to ingest or consume molecular hydrogen, including inhalation of hydrogen gas, drinking hydrogen-dissolved water (hydrogen-rich water) and injecting hydrogen-dissolved saline are preferred approaches. Because inhalation of hydrogen gas or injection of hydrogen-dissolved saline may be unsuitable or impractical for continuous molecular hydrogen consumption in daily life for preventive use, we chose to examine hydrogen-rich water consumption in our present study. Hydrogen-rich water is a particularly promising mean to ingest molecular hydrogen, since it is portable, easily administered, and safe and does not affect the taste, smell, or pH of foods and drinks. Hydrogen-rich water can be obtained by several methods: infusing hydrogen gas into water under high pressure, electrolyzing water to produce hydrogen, and reacting metal or hydride with water. These methods could be useful for application to not only water but also other solvents. Since molecular hydrogen is a very small molecule, it is easily lost and penetrates the glass and plastic walls of any vessel in a short time. Therefore, hydrogen-rich water had to be prepared every day for this study. Consequently, we prepared hydrogen-rich water just before administration and changed the water twice a day in a closed glass vessel equipped with an outlet line containing two ball bearings, which kept the water from being degassed.

Nrf2 is the master regulator of antioxidant response elements and modulates the expression of defense genes associated with protection against injuries and diseases [30]. Activated Nrf2 in the cytoplasm translocates to the nucleus, where it binds to promoters and upregulates the expression of phase 2 enzymes such as HO-1 and NQO-1. Intriguingly, the Nrf2-mediated defense system has been reported to play crucial roles in wound healing in some tissues [27, 31]. Furthermore, HO-1, an anti-inflammatory and antioxidant enzyme, was also suggested to be associated with wound healing in humans [32]. The expression of HO-1 is appreciated to catalyze heme into equimolar quantities of biliverdin (reduced subsequently to bilirubin through biliverdin reductase), which frees ferrous iron and carbon monoxide [33], thereby negatively regulating iNOS through both carbon monoxide-mediated inactivation of iNOS and ferrous iron ion inhibition of iNOS transcription [34]. Recently accumulating evidence suggests that molecular hydrogen promotes the upregulation of Nrf2 in lung tissue [8]. Additionally, hydrogen-induced enhancement of Nrf2 expression significantly increased the expression of HO-1 and NQO-1 [8]. As hydrogen-rich water intake was shown to activate the Nrf2/antioxidant pathway, we measured the expression of Nrf2, HO-1, NQO-1, and iNOS. In our experiment, enhancement of the expression of Nrf2 was observed in the palatal tissue of the rats administered hydrogen-rich water. Moreover, the activation of Nrf2 elevated the expression of HO-1 and NQO-1 and reduced iNOS expression, as determined using real-time PCR and western blotting of palatal tissue samples.

The peripheral blood marker ROM is reported to be useful as a reliable indicator of circulating ROS [35]. Analysis of ROM helps determine the overall oxidant capacity in blood, which in turn reflects the level of ROS from which they were formed. Several studies have indicated the existence of a relationship between systemic levels of ROM and systemic disease states. Because of inflammation, serum levels of ROM also increase in periodontitis patients [36]. Additionally, administration of hydrogen-rich water decreases periodontitis-induced systemic ROM, which in turn inhibits inflammation [26]. It is known that NO and nitrotyrosine levels significantly increase in wounds and wounded tissue [37]. NO, which is produced by nitric oxide synthase through the oxidation of l-arginine, is a secondary messenger involved in various physiopathological processes and is readily oxidized to nitrite (NO_2_). Furthermore, NO_2_ can be oxidized to nitrate (NO_3_). The total levels of nitrite and nitrate in blood are generally used for monitoring NOx [38]. Additionally, peroxynitrite, a highly reactive oxidant formed by the combination of nitric oxide and superoxide, can nitrosylate tyrosine residues of proteins to produce nitrotyrosine residues. Recently, several methods have become available to evaluate total antioxidant status in the blood. In this study, systemic antioxidant capacity was measured by spectrophotometric estimation using the OXY-adsorbent test, which tests the sample's ability to oppose the massive oxidative action of a hypochlorous acid solution [39]. The data from this study showed that oral administration of hydrogen-rich water markedly activated antioxidant capacity and consequently reduced systemic oxidative and nitrosative stresses. It is therefore possible that molecular hydrogen accelerates the wound healing process by inhibiting systemic oxidative and nitrosative stresses.

This study has a limitation. While clinical photographs of palatal wounds were taken with a digital camera, no histological or immunohistochemical analysis using a light microscope was presented. Further detailed investigations, such as histological analyses, for defining how hydrogen-rich water modulates palatal wounds would be needed to improve the reliability of our notion.

## 5. Conclusions

In conclusion, drinking hydrogen-rich water exhibited multiple beneficial effects in the rat palatal wound model through activation of the Nrf2/antioxidant defense pathway. The results of this study support the hypothesis that oral administration of hydrogen-rich water would be beneficial during the wound healing process by decreasing oxidative stress and inflammatory responses.

## Figures and Tables

**Table 1:** Primers used in real-time PCR.

Gene	Orientation	Primer sequence
*Nrf2*	Forward	5′-GCTATTTTCCATTCCCGAGTTAC-3′
	Reverse	5′-ATTGCTGTCCATCTCTGTCAG-3′

*HO-1*	Forward	5′-CTTTCAGAAGGGTCAGGTGTC-3′
	Reverse	5′-TGCTTGTTTCGCTCTATCTCC-3′

*NQO-1*	Forward	5′-CATCATTTGGGCAAGTCC-3′
	Reverse	5′-ACAGCCGTGGCAGAACTA-3′

*iNOS*	Forward	5′-ACCACTCGTACTTGGGATGC-3′
	Reverse	5′-CACCTTGGAGTTCACCCAGT-3′

*IL-1β*	Forward	5′-TGTGATGTTCCCATTAGAC-3′
	Reverse	5′-AATACCACTTGTTGGCTTA-3′

*IL-6*	Forward	5′-CCCAACTTCCAATGCTCTCCTAAT-3′
	Reverse	5′-GCACACTAGGTTTGCCGAGTAGACC-3′

*TNF-α*	Forward	5′-GTGATCGGTCCCAACAAG-3′
	Reverse	5′-ATCGGGTGCAGCATCGTT-3′

*MCP-1*	Forward	5′-CTCTCTTCCTCCACCACTATGC-3′
	Reverse	5′-GTGGGGCATTAACTGCATCTG-3′

*MIP-1α*	Forward	5′-TCCACCACTGCCCTTGCT-3′
	Reverse	5′-CGTCCATAGGAGAAGCAGCA-3′

*TGF-β1*	Forward	5′-CTCCCGTGGCTTCTAGTGC-3′
	Reverse	5′-GCCTTAGTTTGGACAGGATCTG-3′

*FGF7*	Forward	5′-TCTATAATGCGCAAATGGATACTGA-3′
	Reverse	5′-CGAGGTGGAAGCACGGTCT-3′

*VEGF*	Forward	5′-GGGCTGCTGCAATGATGAA-3′
	Reverse	5′-TCCGCATGATCTGCATAGTGA-3′

*α-SMA*	Forward	5′-AGCATCCGACCTTGCTAACG-3′
	Reverse	5′-CATACATGGCAGGGACATTGAA-3′

*Col-1*	Forward	5′-GTGGAAATGATGGTGCTACT-3′
	Reverse	5′-TTAGCACCAGTGTCTCCTTT-3′

*GAPDH*	Forward	5′-GTATTGGGCGCCTGGTCACC-3′
	Reverse	5′-CGCTCCTGGAAGATGGTGATGG-3′
